# Ligand‐Mediated Shape Engineering of SWIR InAs Colloidal Quantum Dots for Photodetector Applications

**DOI:** 10.1002/adma.74004

**Published:** 2026-07-11

**Authors:** Taewan Kim, Jae Taek Oh, Hao Wu, Marta Martos Valverde, Debranjan Mandal, Gerasimos Konstantatos

**Affiliations:** ^1^ ICFO–Insitut De Ciències Fotòniques The Barcelona Institute of Science and Technology Castelldefels Barcelona Spain; ^2^ ICREA‐Institució Catalana de Recerca i Estudis Avançats Barcelona Spain

**Keywords:** InAs quantum dots, shape‐controlled nanocrystal growth, solution‐phase ligand‐exchange, SWIR‐photodetector

## Abstract

Indium arsenide colloidal quantum dots (InAs CQDs) are promising candidates for next‐generation short‐wave infrared (SWIR) optoelectronics; however, synthesis using non‐pyrophoric amino‐arsine precursors has faced limitations in reaching long wavelengths (>1300 nm) due to intrinsic growth stagnation and challenging surface control. Herein, we report a robust strategy to synthesize high‐quality SWIR InAs CQDs by introducing bulky oleic acid (OA) ligands to overcome these kinetic barriers. By modulating the interplay between the steric hindrance of OA ligands and the stabilization of (111) facets, we induce a controlled shape evolution from initial kinetically stabilized spheres to thermodynamically stable tetrahedrons. This morphological transition enables the achievement of the same absorption wavelength followed by a nearly fourfold reduction in nanocrystal volume compared to spherical counterparts. This mechanism offers a distinct pathway for the bandgap tunability of InAs CQDs enabling access to the SWIR region (>1700 nm) under mild reaction conditions (260–280°C). Furthermore, the OA‐mediated surface chemistry unlocks the formulation of conductive halide‐based inks, enabling photodetector development based on amino‐arsine‐synthesized InAs CQDs extending their spectral coverage to 1.6 um. The resulting photodetectors exhibit an external quantum efficiency of 19.1% at 1300 nm and D* of 3.4 × 10^10^ Jones.

## Graphical abstract

The table of contents: A robust ligand‐engineering strategy is introduced for SWIR InAs CQDs synthesized from amino‐arsine precursors. Bulky OA ligands induce controlled shape evolution, allowing access to >1700 nm absorption under mild conditions. Halide‐based inkification enables photodetectors with 19.1% EQE at 1300 nm and detectivity exceeding 10^10^ Jones.

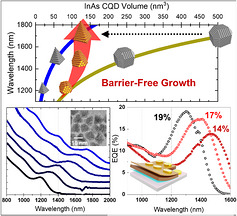

## 1 Introduction

Indium arsenide colloidal quantum dots (InAs CQDs) have garnered significant attention as one of the promising candidates for short‐wave infrared (SWIR) optoelectronic applications because of their tunable bandgap, RoHS compliance, and promising thermal resilience compared to lead‐based counterparts [1]. Historically, the synthesis of high‐quality InAs CQDs has been dominated by methods utilizing tris(trimethylsilyl)arsine ((TMS)_3_​As) and tris‐trimethylgermyl arsine as the arsenic precursor [2, 3, 4, 5]. These group‐14‐substituted organoarsine precursors‐based syntheses typically employ oleate ligands to yield spherical nanoparticles, governed by a diffusion‐controlled growth mechanism. In particular, the continuous injection strategy has proven its effectiveness by achieving size uniformity of resulting CQDs via size focusing and achieving high chemical yields, allowing precise control over CQD size and bandgap under fixed reaction conditions [3, 5]. Consequently, high‐performance devices based on these spherical InAs CQDs have been widely reported, yet primarily in the NIR region [6, 7, 8, 9, 10]. However, the prohibitively high cost and high pyrophoricity of (TMS)_3_As pose severe challenges to the large‐scale deployment and industrial adoption of SWIR InAs CQD devices.

To address these economic and safety concerns toward scalability, synthetic routes employing stable amino‐arsine (amino‐As) precursors have emerged as a compelling alternative [11, 12]. The amino‐As‐based synthesis typically utilizes oleylamine (OLAm) and chloride (Cl^–^) as ligands, resulting in the formation of tetrahedral nanocrystals of which (111) facets are bounded by OLAm and Cl^–^. While amino‐As precursors offer superior cost‐competitiveness and demonstrate the capability to synthesize large‐sized InAs CQDs comparable to those from TMS_3​_As, this synthesis faces distinct challenges. The synthesis is often highly sensitive to reaction parameters, requiring delicate fine‐tuning of conditions such as reaction time and temperature for controlling the size of CQDs [11, 12, 13, 14]. For example, a recent study on amino‐As precursors applied continuous temperature ramps to achieve 1600 nm wavelength regime [14]. Furthermore, a recent study employing the In(I)Cl and amino‐As complex demonstrated that carrying out the reaction at 300°C for a duration of several hours yields large InAs particles that approach bulk dimensions [15].

Another limitation lies in their surface chemistry; the OLAm and Cl^–^ ligands are strongly bound to the surface of InAs CQDs, thereby significantly hindering efficient ligand exchange processes and conductive ink formulation [16]. Consequently, despite the economic advantages of the precursors, reports on photodiodes utilizing amino‐As‐based InAs CQDs remain scarce compared to their (TMS)_3_​As counterparts [17, 18]. To date, surface functionalization has been predominantly restricted to thiol‐based ligand exchanges, and achieving high‐efficiency devices combined with fast response speed beyond 1300 nm remains a challenge.

The exact mechanism driving the preferential formation of tetrahedral morphologies in amino‐As syntheses is likely attributed to the high stability of (111) facets passivated by chloride and amine ligands, consequently requiring high reaction temperatures [11, 15, 19]. Such faceted growth behavior implies that the synthesis likely proceeds via a reaction‐controlled mechanism, analogous to observations in other faceted nanocrystal systems. For instance, for the synthesis of tetrahedral CdSe nanoparticles, the reaction time or temperature required for particle growth increases exponentially with larger facets [20, 21]. Similarly, other faceted nanoparticles typically demand elevated temperatures or significantly extended reaction durations to sustain growth [22, 23, 24]. Drawing parallels to these systems, we anticipated that amino‐As‐based InAs synthesis encounters similar intrinsic kinetic barriers when targeting large‐sized CQDs.

Herein, we report a robust strategy for synthesizing high‐quality large‐size InAs CQDs utilizing amino‐As precursors in an oleic acid (OA) and trioctylphosphine (TOP) coordination environment. By introducing bulky OA ligands with relatively weak surface binding affinity, we successfully induce a diffusion‐controlled growth regime, promoting the initial formation of spherical InAs nanocrystals. Notably, by carefully modulating the surface energy and reaction conditions, we demonstrate a controlled morphological evolution from kinetically stabilized spheres to thermodynamically stable truncated tetrahedrons, which enables the CQDs to readily extend their absorption cutoff beyond 1700 nm. This unique shape transition effectively bypasses the high activation energy barriers typically associated with continuous faceted growth, allowing for facile access to the SWIR region under mild temperature conditions (260°C–280°C) and short reaction times (<1 h), both being highly determinant factors for a scalable process. Furthermore, the OA‐rich surface chemistry unlocks the formulation of conductive, halide‐passivated, InAs CQD inks, distinguishing our approach from conventional amino‐As‐based systems that have predominantly relied on thiol‐mediated solid‐state ligand exchanges [17, 18, 19]. Leveraging these material advancements, the fabricated SWIR photodetectors exhibit a high external quantum efficiency (EQE) of 19.1% and a reverse dark current density of 14.6 uA/cm^2^ at –1 V, validating the efficacy of this cost‐effective route toward high‐performance optoelectronics.

## 2 Results and Discussion

### 2.1 Synthesis of Oleic Acid‐Based InAs CQDs

**FIGURE 1:**
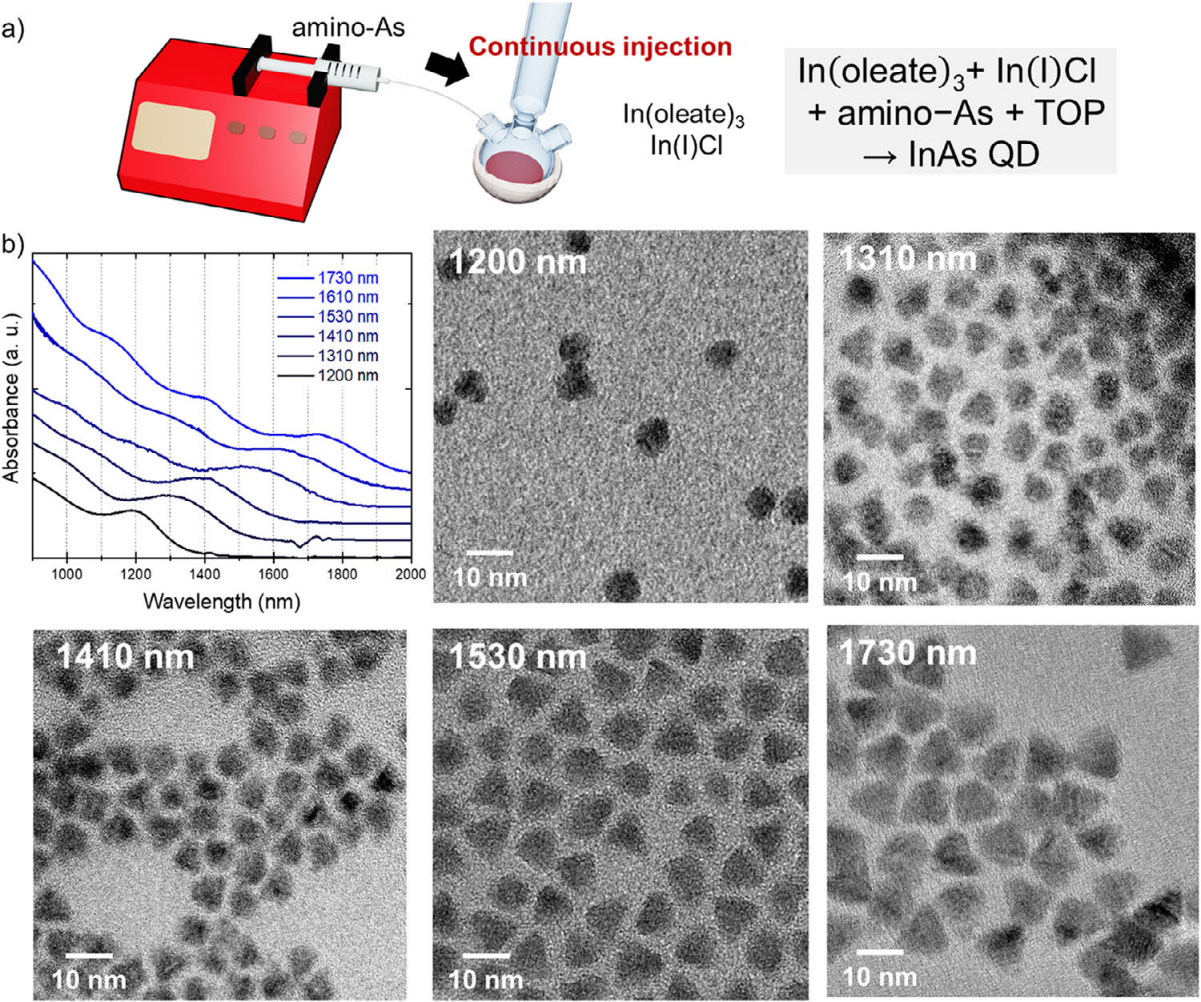
Synthesis strategy and morphological evolution of SWIR InAs CQDs. (a) Schematic illustration of the continuous injection synthesis designed to access the SWIR region. (b) Evolution of UV‐vis‐NIR absorption spectra controlled by the precursor ratio and reaction time, along with TEM images corresponding to specific absorption peaks (1200 nm, 1310 nm, 1410 nm, 1530 nm, and 1730 nm). The TEM images reveal a distinct morphological transition from spherical (∼1200 nm) to faceted tetrahedral geometries (∼1730 nm) as the particle size increases.

Indium(I) chloride (In(I)Cl) was selected as the reducing agent because of its mild reducing power and proven capability to ensure high chemical yields in amino‐As systems [11]. Unlike conventional protocols that rely solely on OLAm and Cl^–^, which often leads to growth stagnation due to strong facet passivation, we hypothesized that the employment of OA into the reaction would offer steric hindrance reducing thereby the activation energy for growth. This destabilized surface environment is essential to fully leverage the continuous injection method (Figure 1a), enabling the sustained growth of CQDs into the SWIR region (see Experimental Section for details). In addition, to achieve a distinct separation between nucleation at low temperature and growth, we adapted a previously reported temperature‐ramping method [14].

Figure 1b presents the evolution of InAs CQD absorption peaks as a function of the seed‐to‐growth precursor solution ratio and reaction temperature (see Experimental Section for details). By controlling these reaction conditions, we successfully achieved InAs CQDs with a first excitonic peak extending beyond 1700 nm. X‐ray diffraction (XRD) patterns confirm that the zinc blende crystal structure is preserved throughout the growth process (Figure S1). Furthermore, statistical analysis of the transmission electron microscopy (TEM) images demonstrates that a narrow size distribution is maintained across the size ranges (Figure S2). Interestingly, (TEM) imaging reveals a distinct morphological evolution accompanying this spectral shift. As the precursor ratio and reaction temperature change, the initially spherical InAs CQDs (observed at ∼1200 nm) onset a transition toward a tetrahedral geometry as shown in Figure 1b. This growth behavior contrasts sharply with conventional amino‐As‐based syntheses, which typically yield tetrahedral nanocrystals from the early stage. Furthermore, achieving such large‐sized CQDs at moderate temperatures (260^°^C–280^°^C) is intriguing, considering that conventional amino‐As routes typically require temperatures exceeding 300^°^C to drive growth beyond 1300 nm [11]. Notably, this protocol maintains a chemical yield of approximately 60% for CQDs absorbing below 1400 nm, and retains a yield of ∼30% even for larger CQDs absorbing above 1500 nm.

### 2.2 Growth Kinetics and Morphological Evolution of InAs CQDs

**FIGURE 2:**
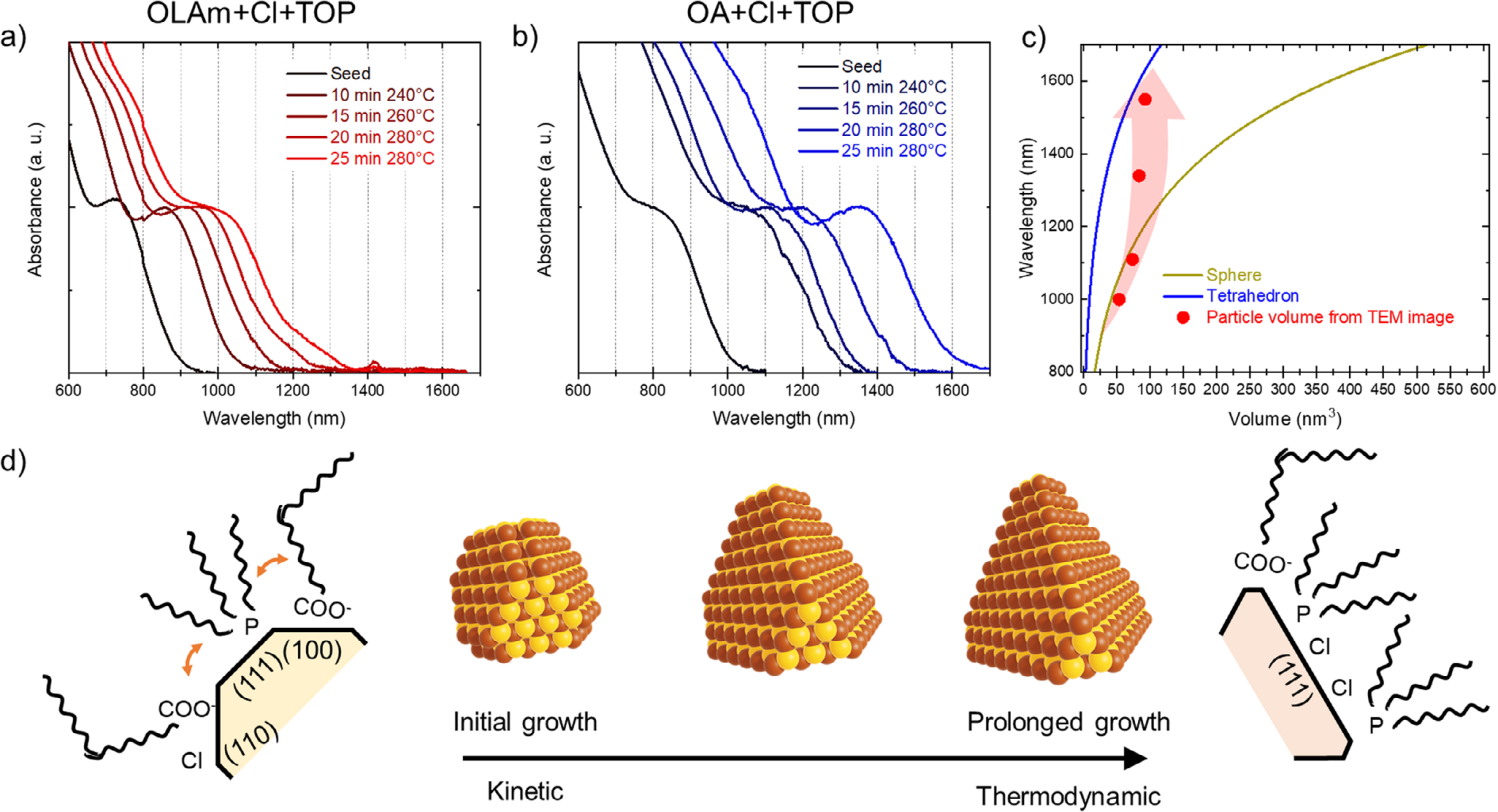
Ligand‐dependent growth kinetics and morphological evolution mechanism of amino‐As‐based InAs CQDs. (a,b) Temporal evolution of UV‐vis‐NIR absorption spectra for InAs CQDs synthesized with (a) OLAm+Cl and (b) OA+Cl+TOP as the primary ligands. Under identical reaction conditions, the OLAm‐based system exhibits growth stagnation near 1000 nm (tail to 1200 nm), whereas the OA‐based system demonstrates a sustained spectral redshift extending beyond 1350 nm. (c) Correlation between the first excitonic peak wavelength and nanocrystal volume. The solid olive and blue lines represent the theoretical confinement trajectories for spherical and tetrahedral geometries, respectively. The experimental data (red symbols) trace a morphological transition: starting from the spherical regime (1000 nm and 1110 nm), the CQDs converge onto the truncated tetrahedral trajectory (1340 and 1550 nm). (d) Schematic illustration of the proposed growth mechanism. The steric hindrance imposed by bulky OA and TOP ligands initially promotes kinetically controlled isotropic growth (quasi‐spherical). As the reaction proceeds, the system transitions to a thermodynamically favored regime, driving the morphological evolution into tetrahedrons.

To further corroborate our hypothesis on how the introduction of bulky oleate ligands alters the surface chemistry and consequent growth kinetics of amino‐As‐based InAs CQDs, we also performed synthesis based on OLAm. Figure 2a,b display the temporal evolution of absorption spectra for InAs CQDs synthesized with OLAm and OA as the primary long‐chain ligands, respectively. To ensure a rigorous comparison, all experimental parameters—including the reducing agent (In(I)Cl), precursor concentration, and reaction temperature—were kept strictly identical, with the exception of the ligand environment (OA in ODE vs. OLAm; see Experimental Section). In both reaction systems, nucleation occurred immediately upon injection, forming initial seeds absorbing at approximately 700–800 nm. However, a striking divergence in growth behavior was observed. In contrast to the OLAm‐mediated system, which exhibited growth stagnation near 1020 nm (with only a negligible tail extending toward 1240 nm) over the course of 25 min, the OA‐based CQDs demonstrated a significantly more rapid and sustained spectral redshift, extending well beyond 1350 nm under the same thermal and concentration profiles. Quantitative analysis of the spherical CQD volume up to 1200 nm in Figure 2b reveals a linear growth rate of approximately 3.4 nm^3^ min^−^^1^, directly corresponding to the constant precursor injection. This linear volume expansion, accompanied by the progressive sharpening of absorption peaks, indicates that the InAs CQDs follow a diffusion‐controlled growth mechanism within this size regime [5].

To experimentally validate the shape transition and its associated growth mechanism, we tracked the morphological evolution of InAs CQDs by terminating the reaction at different time intervals under identical synthetic conditions. Figure S3 displays the absorption spectra of the crude solutions without size selection alongside their corresponding high‐resolution TEM images. Nanocrystals exhibiting first excitonic peaks at 1000 nm and 1110 nm displayed multi‐faceted geometries. As the reaction progressed and the absorption shifted to 1340 nm and 1550 nm, the particles shifted into truncated tetrahedral structures. Figure 2c presents the nanocrystal volumes determined by TEM alongside the theoretical Bohr equations for spherical and tetrahedral InAs CQDs. The volumetric ratios calculated from the TEM measurements demonstrate a strong correlation with the optical density ratios obtained from the absorption spectra. Furthermore, TEM analysis confirmed that the InAs CQDs with a first excitonic peak at 1730 nm also exhibit a truncated tetrahedral morphology, as shown in Figure S3. As the nanocrystals grow, the relative extent of this truncation appears to decrease gradually. This experimental agreement indicates that the spectral shift originates not only from the increased size but also from a morphological transition from spherical to tetrahedral geometries. Consequently, this morphological transition reduces the volume expansion required during nanocrystal growth. For example, while previously reported spherical InAs CQDs with an absorption peak at 1600 nm possess a volume of approximately 400 nm^3^ [5], the tetrahedral particles demonstrate a volume of approximately 100 nm^3^ [12].

We attribute this distinct growth behavior and the presence of truncated tetrahedral shape in large size particles primarily to the steric hindrance imposed by bulky OA and TOP ligands, which destabilizes the surface facet [25]. In conventional amino‐As protocols, the (111) facets are stabilized by a compact ligand shell of Cl^–^ and OLAm (typically in a 3:1 ratio) [26]. In contrast, the coexistence of bulky OA, TOP, and Cl^–^ in our system sterically impedes the exclusive passivation of (111) facets, promoting the formation of isotropic, multi‐faceted (quasi‐spherical) nanocrystals in the early stages, as shown in Figure 2d.

To validate this hypothesis, we performed a control experiment using only OA and TOP without any chloride source, introducing the 1,1,3,3,5,5‐hexamethyltrisiloxane (HMTS). As shown in Figure S4 and reported previously, InAs CQDs synthesized with HMTS showed tetrahedral shape [18]. However, the resulting nanocrystals with HMTS reducing agent with OA and TOP ligand exhibited an even more spherical morphology, suggesting that bulky ligands drive isotropic growth in the absence of facet‐selective halide.

By leveraging this sphere‐to‐tetrahedron transition, our strategy efficiently accesses the SWIR region overcome 1700 nm at moderate temperatures and faster rates, effectively bypassing the high volumetric and energetic barriers that typically hinder the continuous growth of spherical particles in conventional synthesis.

### 2.3 Ink Formulation of InAs CQDs

**FIGURE 3:**
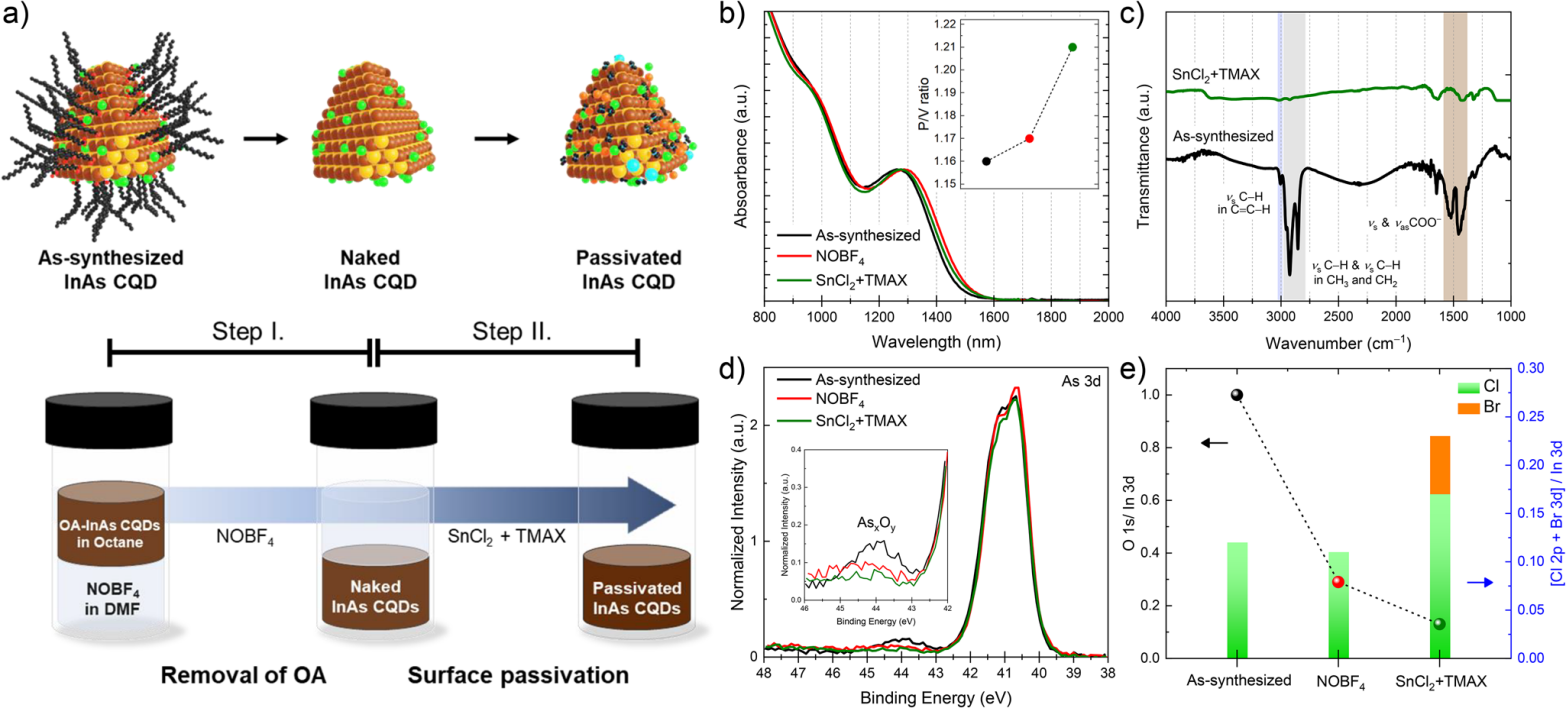
Inkification strategy and its impact on the optical properties and surface chemistry of InAs CQDs. a) Schematic illustration of the two‐step inkification process for InAs CQDs. b) UV–vis absorption spectra of as‐synthesized InAs CQDs (black) and inkified InAs CQDs obtained via the two‐step process using NOBF_4_ (red) and SnCl_2_ + TMAX (olive). The inset shows the peak‐to‐valley (P/V) ratio of InAs CQDs as a function of the inkification process c) FT‐IR spectra of as‐synthesized (black) and inkified (olive) InAs CQDs films. Symbols of *ν*_s_ and *ν*_as_ represent vibrational modes of symmetric and asymmetric stretches, respectively. d) As 3d XPS spectra of as‐synthesized InAs CQDs (black) and inkified InAs CQDs processed with NOBF_4_ (red) and SnCl_2_ + TMAX (olive). The inset presents an enlarged view of the As_x_O_y_‐related region. e) Relative elemental ratio of oxygen (left) and halides [Cl+Br] (right) species, normalized to In 3d, obtained from XPS measurements of InAs CQDs films.

Following the establishment of a controlled synthetic pathway and growth regime, we next turn our attention to the inkification of the as‐synthesized InAs CQDs via solution‐phase ligand exchange, a critical step toward scalable and high‐performance solution‐processable device fabrication [27]. This process is designed to reconstruct the surface chemistry of InAs CQDs from an insulating, organic‐ligand‐terminated state into an electronically active CQD solid suitable for charge transport. Figure 3a schematically illustrates the inkification process. To formulate InAs CQD inks, we employed a two‐step, sequential inkification strategy. In the first step, OA‐capped InAs CQDs were treated with nitrosonium tetrafluoroborate (NOBF_4_) to remove native oleate ligands as well as residual metal oxides from the CQD surface. This ligand‐stripping process exposes undercoordinated surface sites and facilitates the removal of insulating organic species, thereby preparing a chemically reactive surface for the following passivation step [28, 29]. Subsequently, a second passivation step was carried out using tin(II) chloride (SnCl_2_), which exhibited high affinity for surface As atoms [9], together with tetramethylammonium halides (TMAX) acting as Z‐ and X‐type ligands to passivate surface dangling bonds, neutralize charged defect states, and stabilize the CQDs for ink formulation.

Figure 3b presents the UV–vis absorption spectra of InAs CQDs before and after the inkification process. After the two‐step inkification, the excitonic peak of the InAs CQDs becomes more pronounced. As shown in the inset of Figure 3b, the increase in peak‐to‐valley (P/V) ratio of InAs CQDs as a function of inkification process indicates improved suppression of surface‐related trap‐states resulting from the SnCl_2_ and TMAX treatment. Fourier transform infrared (FT‐IR) spectroscopy further supports the efficient removal of native OA ligands after the inkification process, as shown in Figure 3c. Specifically, the disappearance of the COO^−^ symmetric and asymmetric stretching vibration at 1525 cm^−^^1^, together with the symmetric C─H stretching modes of the alkyl chains (CH_3_ and CH_2_) at 2854, 2924, and 2956 cm^−^^1^, as well as the symmetric C═C stretching vibration at 3005 cm^−^^1^, confirms the effective exchange of oleate ligands. To further investigate the impact of the inkification process on the surface chemistry of InAs CQDs, X‐ray photoelectron spectroscopy (XPS) measurements were performed. Figure 3d shows the As 3d spectra acquired before and after the sequential inkification process. For the oleate‐based synthesis, the presence of arsenic oxide (As_x_O_y_) species—commonly associated with deep trap states arising from high reaction temperatures during synthesis [30]—is clearly observed prior to inkification at the higher binding energy region. Notably, this peak is progressively suppressed after inkification, indicating substantial removal of As_x_O_y_ from the InAs CQDs surfaces. Consistent with this observation, Figure 3e presents the relative elemental ratios of oxygen and halide species with respect to indium in InAs CQDs films. Following the two‐step inkification process with SnCl_2_ + TMAX, the oxygen content decreases from 1.00 to 0.13 while the halide content increases from 0.12 to 0.23, suggesting a substantial reduction of surface metal oxides accompanied by effective halide passivation of the InAs CQDs surfaces (Figure S5). These results confirm that the inkification process induces an efficient surface ligand exchange while simultaneously removing native organic ligands and surface oxides. This effective surface chemical reconstruction provides a trap‐suppressed InAs CQDs ink suitable for subsequent thin‐film processing and high‐performance device fabrication.

### 2.4 SWIR InAs CQD Photodiodes

**FIGURE 4:**
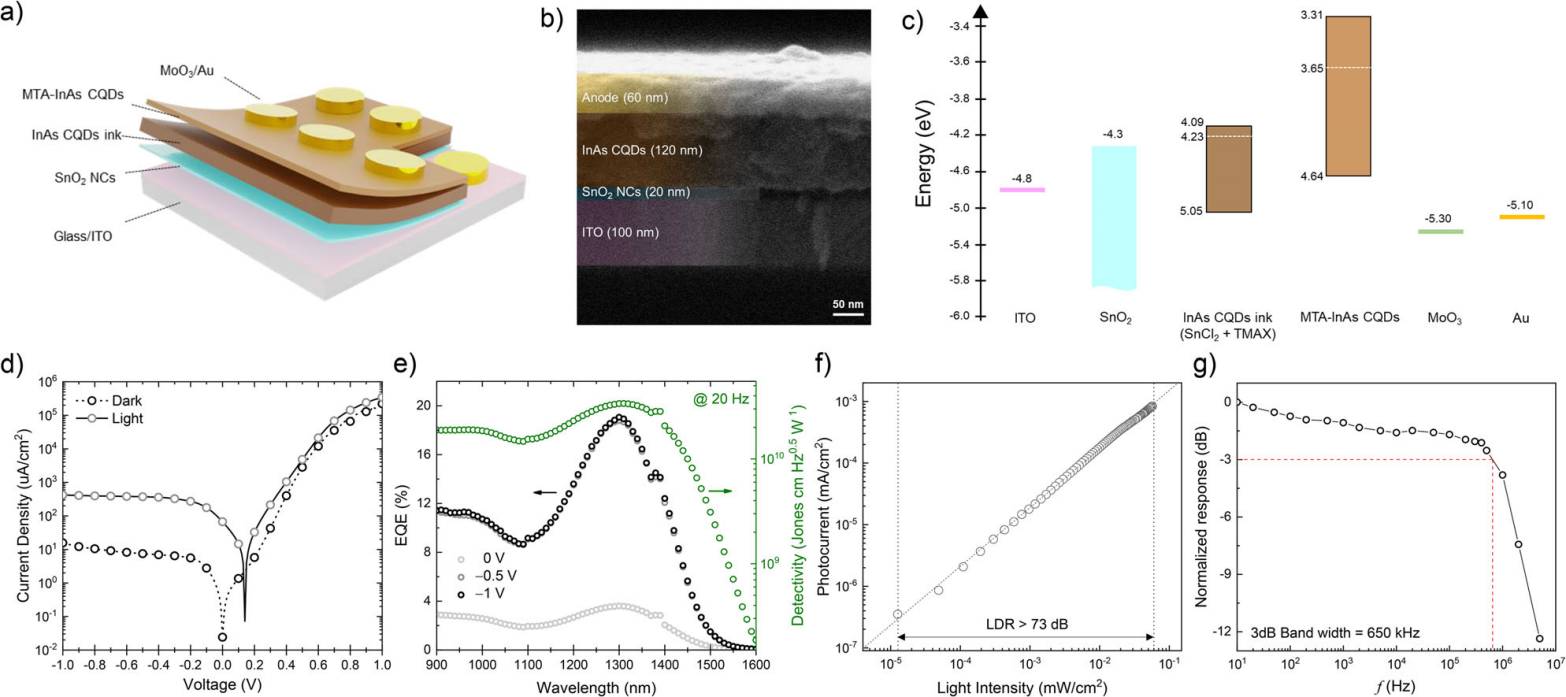
Device performance of InAs CQDs short‐wave infrared (SWIR) photodetectors. a) Schematic illustration of the device architecture employed for the InAs CQD photodetectors. b) Cross‐sectional SEM image of InAs CQDs photodetector. c) Energy level diagram of the InAs CQDs photodetector extracted from ultraviolet photoelectron spectroscopy (UPS) measurements, showing the relative band alignment of the InAs CQD layer and charge transport layers. d) Current density–voltage (*J–V*) characteristics of the InAs CQD photodetector in dark and under 1300 nm illumination (intensity ∼3 mW cm^−2^). e) External quantum efficiency (EQE) spectra (black, left) and specific detectivity spectrum (olive, right, at 20 Hz and 0 V, in jones) of the InAs CQDs photodetector. f) Linear dynamic range (LDR) of InAs CQDs photodetector under 1310 nm light. g) Frequency response bandwidth of InAs CQDs photodetector.

Building on the inkification‐driven surface chemical reconstruction, we next investigated the optoelectronic performance of InAs CQDs photodetectors. Figure 4a shows the device architecture of the InAs CQDs photodetector with an ITO/Tin(IV) oxide nanocrystals (SnO_2_ NCs)/InAs CQDs ink/MTA–InAs CQDs/MoO_3_/Au configuration. Figure 4b presents a cross‐sectional scanning electron microscopy (SEM) image of the InAs CQDs photodetector, revealing an InAs CQDs layer thickness of approximately 120 nm. Figure 4c presents the energy level alignment of InAs CQDs photodetector. To form an improved junction between the InAs CQDs ink layer and the MoO_3_/Au anode, small‐sized InAs CQDs (Figure S4) exchanged with 2‐methoxyacetic acid (MTA), previously reported in the literature as a ligand capable of lowering the energy levels of InAs CQDs film, were introduced as an electron‐blocking interlayer (Figure S6) [31]. This energy‐level alignment promotes efficient hole extraction while suppressing electron injection at the MoO_3_/Au interface, thereby reducing leakage current under reverse bias and enabling strong rectifying behavior (Figure S7).

Figure 4d displays the *J–V* characteristics of the InAs CQD photodiode fabricated using the 1280 nm InAs CQDs ink presented in Figure 3b. The device exhibits a dark current density of approximately 15.7 µA cm^−2^ at −1.0 V, along with a rectification ratio exceeding 10^4^ at ±1 V, indicative of efficient diode operation. The corresponding device optimization process focused on the type of alkylammonium halides and the halide composition used in the inkification are presented in Figure S8, S9. We also fabricated a device based on InAs CQDs ink using indium(III) bromide and ammonium bromide (InBr_3_ and NH_4_Br) as passivating agents, modified from a previously reported method [7]. However, this device exhibited poor rectifying behavior as a photodiode. This can be attributed to the excessively deep band‐edge positions of the InBr_3_ + NH_4_Br‐passivated InAs CQDs film, which result in unfavorable barrier formation at the heterojunction, ultimately leading to inefficient charge extraction and increased leakage current (Figure S10). Figure 4e shows the EQE spectra of the InAs CQDs ink photodetector, with a maximum EQE of 19.1% at 1300 nm measured under a reverse bias of −1 V. To the best of our knowledge, this value represents the highest reported EQE at 1300 nm among SWIR photodetectors based on non‐pyrophoric As precursor synthesized InAs CQDs (Figure S11). The frequency‐dependent current noise spectrum of the InAs CQDs photodetector was measured with a lock‐in–amplifier–based setup, where a noise current of ∼1.99 × 10^−13^ A Hz^−0.5^ was recorded at a frequency of 20 Hz (Figure S12). Therefore, the InAs CQDs photodetector exhibited a specific detectivity (D*) ∼3.4 × 10^10^ jones at 20 Hz at 1300 nm. The frequency‐dependent specific detectivity of InAs CQD photodetector is presented in Figure S13. To further assess the generality of the inkification strategy, we prepared InAs CQD inks and fabricated photodetector devices using InAs CQDs with different sizes, corresponding to first excitonic peaks at 1360 and 1415 nm (Figure S14, S15). Altering the CQD size or their shape inevitably induces changes in surface chemistry passivation and thereby optoelectronic properties, rendering definitive assignment on the role of each on device behavior a difficult task. Despite these complex interdependencies, the results show that maximum EQE values of 17.3% and 13.9% were achieved at 1410 and 1470 nm, respectively, which are also among the highest reported values for amino‐As‐synthesized InAs CQD–based photodetectors with corresponding dark current densities of 40.9 and 194.8 µA cm^−2^ at −1.0 V, indicating that our inkification process remains effective across a wide range of InAs CQD sizes. Collectively, these results demonstrate that the proposed inkification strategy and device architecture provide a viable route toward InAs CQD photodetectors with promising performance metrics in the >1100 nm regime, as summarized in Table 1.

**TABLE 1:** Comparison of this work with state‐of‐the‐art InAs CQD photodetectors operating beyond 1100 nm in terms of supplier, arsenic source, InAs CQD type, ligand‐exchange method, device structure, wavelength, dark current density, EQE, detectivity, and response time.

Material (supplier)	Arsenic source	Type	Ligand exchange method (Ligand type)	Device structure	Wavelength [nm]	Dark current density [µA/cm^2^] at −1 V bias	EQE [%] at bias [V]	Detectivity [jones] at bias [V]	Response time (µs) at bias [V]	Refs.
In(As,P) CQD (lab‐synthesized)	amino‐As	Core	Solution‐phase (amine and thiol)	ITO/NiO/In(As,P) CQD/TiO_2_/Al	1140	0.85	3.37 at −3	1.1 × 10^10^ at −3^a^	∼0.1 at −3^b^	[17]
					1270	1.54	2.83 at −3	6.11 × 10^9^ at −3^a^		
					1400	1.74	0.62 at −3	1.01 × 10^9^ at −3^a^		
InAs CQD (lab‐synthesized)	TMS_3_As	Core	Solution‐phase (halide)	ITO/ZnO/InAs CQD/MoO_3_/Ag	1120	0.4	35 at −1	N/A	1.5 at −1	[31]
InAs/ZnSe CNQD (lab‐synthesized)	AsI_3_	Core/ shell	Solid‐state (thiol)	ITO/ZnO/InAs CNQD/MoO_3_/Au	1450	∼20	∼14 at −1	∼1.2 × 10^10^ at −1 (>10 Hz)	1.06 at −1	[32]
InAs CQD (lab‐synthesized)	amino‐As	Core	Solid‐state (thiol)	ITO/ZnO/InAs CQD/MoO_3_/Au	1310	570	7.6 at −1	6 × 10^9^ at −1^a^	7.0 at −1	[18]
InAs CQD (lab‐synthesized)	TMS_3_As	Core	Solution‐phase (halide)	ITO/ZnO/InAs CQD/MoO_3_/Ag	1140	0.24	75 at −1	5.0 × 10^11^ at −1 (>20 kHz)	1.0 × 10^−2^ at N/A	[33]
InAs CQD (purchased)	N/A	Core	Solution‐phase (halide)	ITO/MoO_3_/PTB7‐Th/CuI/InAs CQD/IGZO/TiN/Si	1210	4.7	25 at −1	∼2 × 10^10^ at −1 (at 1 Hz)	∼6.5 at N/A	[34]
InAs CQD (lab‐synthesized)	TMS_3_As	Core	Solution‐phase (Thiol & halide)	ITO/ZnO/InAs CQD/MoO_x_/Ag	1500	∼8.0	22 at −0.15	5.2 × 10^11^ at −0.15^a^	N/A	[35]
				ITO/NiO_x_/InAs CQD/PCBM/BCP/Ag		40	∼30 at −1	1.0 × 10^11^ at −1^a^	∼0.2 at N/A	
InAs CQD (lab‐synthesized)	amino‐As	Core	Solution‐phase (halide)	ITO/SnO_2_/InAs CQD/MoO_3_/Au	1300	15.7	19.1 at −1	3.37 × 10^10^ at 0 at 1300 nm (at 20 Hz)	0.5 at 0	This work
					1410	40.9	17.3 at −1			
					1470	194.8	13.9 at −1			

^a^ Shot‐noise‐limit theoretical D*;

^b^ Measured with illuminated by a white LED.

To further quantify the device performance, we evaluated the LDR of the InAs CQDs photodetectors by tuning the incident light intensity (Figure 4f). The photocurrent of InAs CQDs photodetector exhibits a linear dependence on optical power over several orders of magnitude, yielding over 73 dB. The temporal response of the InAs CQDs photodetectors was characterized by measuring the frequency‐dependent photoresponse under modulated illumination. As shown in Figure 4g, the InAs CQDs photodetector showed 3 dB bandwidth of 650 kHz. The transient response the InAs CQDs photodetector under pulsed illumination exhibits a falling time and rising time of 464 ns and 498 ns, respectively (Figure S16). Last, we evaluated the device operational stability in an ambient environment (without any encapsulation); the device maintained its initial output after 26 h (Figure S17). While further optimization of passivation and encapsulation is required to warrant long‐term operational stability for practical industrial applications, these preliminary results demonstrate the potential of our InAs CQD ink‐based SWIR photodetectors.

## 3 Conclusion

In conclusion, we have demonstrated a novel synthetic pathway that overcomes the kinetic limitations of InAs CQD growth within the stable amino‐As precursor system via ligand‐mediated shape engineering. The introduction of bulky OA ligands destabilizes the surface energy during the initial growth stage to induce sustained growth, while the subsequent transition to a thermodynamic truncated tetrahedral morphology serves as a key mechanism enabling extension into the SWIR region (>1700 nm). In particular, our analysis of the shape‐dependent growth trajectory provides critical insights into overcoming the intrinsic volumetric limits of colloidal nanocrystals and offers an additional pathway to bandgap tunability, beyond size tuning, based on shape engineering. Furthermore, the resulting OA‐capped InAs CQDs were effectively processed into conductive halide inks compatible with amino‐As‐based synthetic routes. The fabricated photodetectors demonstrated optoelectronic performance, including a high LDR (>73 dB) and fast response speed. By presenting a safe and economic alternative to high‐cost, high‐risk conventional synthesis methods, this work contributes to accelerating the industrial commercialization of high‐performance InAs CQD SWIR optoelectronic devices.

## 4 Experimental Section

### 4.1 Chemicals

Indium acetate (In(OAc)_3_, 99.99%), 1‐octadecene (ODE, 90%), oleic acid (OA, 90%), 1,1,3,3,5,5‐hexamethyltrisiloxane (HMTS, 95%), N,N‐dimethylformamide (DMF,99.8%), Nitrosyl tetrafluoroborate (NOBF_4_, 95%), tin(II) chloride (SnCl_2_, 99.99%), tetramethylammonium chloride (TMACl), tetramethylammonium bromide (TMABr) and toluene (anhydrous, 99.8%) were purchased from Sigma‐Aldrich. Butanol (99.9%) and Methoxyacetic acid (MTA, 97+%) were purchased from Thermal Scientific. Indium(I) chloride (In(I)Cl, 99.995%) was purchased from Apollo Scientific. Tris(dimethylamino)arsine (amino‐As) was purchased from DOCKWEILER CHEMICALS. Tri‐n‐octylphosphine (TOP, 97%) was purchased from STREM. Oleylamine (OLAm, 80‐90%) was purchased from BIOSYNTH. Dimethyl sulfoxide (DMSO) was purchased from VWR. Acetonitrile and Hexane were purchased from Scharlab. Tin(IV) oxide (SnO_2_) colloidal dispersion (15% in H_2_O) were obtained from Alfa Aesar.

### 4.2 Synthesis of OA‐Based InAs CQDs

To prepare the In‐oleate precursor, a mixture of In(OAc)_3_​ (440 mg, 1.5 mmol), OA (1.6 mL), and ODE (12 mL) was degassed at 110°C for >2 h under vacuum. In(I)Cl (225 mg, 1.5 mmol) was added, and the mixture was degassed for an additional 10 min, backfilled with Ar, and heated to 200°C. The As precursor solution was prepared by dispersing amino‐As (90 µL, 0.5 mmol) in TOP (1 mL). This solution was divided into seed and growth aliquots according to the target CQD size. The synthesis was initiated by the hot injection of the seed solution at 200°C. Following nucleation, the temperature was increased to 240°C. Subsequently, the growth solution was continuously injected at a rate of 1.5 mL/h, while the reaction temperature was gradually raised to 260–280°C.

The chemical yield of the synthesized InAs CQDs was calculated relative to the amino‐As precursor using the previously reported unit cell calculation method based on optical absorbance [5]. The yield ranged from 55% to 65% for particles with absorption peaks shorter than 1400 nm with a typical synthesis providing ∼110 mg. This synthetic procedure maintained nearly identical yield during a twofold scale‐up yielding ∼210 mg per batch. For further scale‐up syntheses, additional investigations into the consistency of the solution stirring rate and heating rate are required.

For the Cl‐free control synthesis, a mixture of In(OAc) _3_​ (440 mg, 1.5 mmol), OA (1.6 mL), and ODE (6 mL) were degassed at 110°C for >2 h under vacuum, backfilled with Ar, and heated to 250°C. The As precursor solution was prepared by dispersing amino‐As (90 µL, 0.5 mmol) in HMTS (1.2 mL). This solution was divided into seed and growth aliquots according to the target CQD size. The synthesis was initiated by the hot injection of the seed solution at 250°C. Subsequently, the growth solution was continuously injected at a rate of 1.2 mL/h. These HMTS‐synthesized spherical nanocrystals were utilized as the small‐sized InAs CQDs for the electron‐blocking interlayer.

All washing steps were performed inside a glove box. The synthesized InAs CQDs were purified using toluene and butanol [5, 8, 12]. First, the crude solution was precipitated with an excess of butanol to remove the solvent. Then, toluene and butanol were mixed at a 1:1 ratio, and the mixture was centrifuged at 6000 rpm for 5 min. During this process, byproducts such as indium oxide and agglomerated CQDs, which can adversely affect device efficiency [36], are removed. Afterward, depending on the size, the CQDs were precipitated at a ratio of 1:1 to 1:4 and redispersed in octane.

### 4.3 Synthesis of OLAm‐Based Control InAs CQDs

To substitute the OA and ODE mixture, 13.6 mL of OLAm was used. In(OAc)_3_ was not used in this process. The solvent was degassed at 110°C for >2 h under vacuum. Subsequently, the synthesis followed the identical protocol to the OA‐based synthesis described above.

### 4.4 Inkification of InAs CQDs

The inkification of InAs CQDs was carried out under N_2_ atmosphere. 2 mL of oleate‐capped InAs CQDs solution dispersed in octane (20 mg mL^−^^1^) was brought into contact with 5 mL of a 0.5 M NOBF_4_ solution in anhydrous DMF, forming a biphasic system. The mixture was vortexed for 2 min to induce phase transfer of the InAs CQDs from the octane phase to the polar DMF phase. After completion of the phase transfer, the upper octane phase was removed. The InAs CQDs solution was washed by adding 5 mL of anhydrous hexane, followed by vortexing for 1 min and removal of the hexane phase. This washing step was repeated three times to eliminate residual organic species. Subsequently, 5 mL of toluene was added, and the naked InAs CQDs were precipitated by centrifugation at 6000 rpm for 5 min. The supernatant was discarded, and the precipitate was redispersed in 2 mL of a DMF/DMSO mixed solvent (1:1, v/v).

For ligand exchange, the naked InAs CQDs solution was mixed with 2 mL of a DMF/DMSO (1:1, v/v) solution containing 50 mM SnCl_2_ and 50 mM tetramethylammonium halide (TMACl:TMABr = 1:1), followed by vortexing for 1 min. The InAs CQDs were then precipitated by adding 4 mL of toluene and finally redispersed in DMF/DMSO containing 1 vol% butylamine, yielding an InAs CQDs ink (175 mg/mL).

### 4.5 Material Characterization

Most of the material characterization followed procedures similar to our previous report [36]. UV–vis absorption measurements were carried out using a Cary 5000 UV–vis–NIR spectroscope in solution. The XRD measurements were performed with a Rigaku SmartLab powder diffractometer with Cu Kα radiation using the Bragg–Brentano geometry. The TEM was performed at the Scientific and Technological Centres of the University of Barcelona. TEM images were acquired using a JEOL 2010F microscope operating at an accelerating voltage of 200 kV, with samples prepared by drop‐casting a diluted CQD solution onto ultrathin carbon grids. XPS/UPS data were collected at the Institut Català de Nanociència i Nanotecnologia using a SPECS PHOIBOS 150 hemispherical analyzer. The system operated under an ultrahigh‐vacuum environment (10^−^^10^ mbar) in conjunction with a 1,486.74 eV monochromatic Kα X‐ray source.

### 4.6 Device Fabrication

The photodetector fabrication was carried out in N_2_‐filled glovebox. ITO‐covered glass substrates (Universität Stuttgart, Institut für Großflächige Mikroelektronik) were cleaned sequentially by sonication in soapy water, acetone, and isopropanol for 30 min each and dried with nitrogen blowing. After UV‐ozone treatment for 30 min, SnO_2_ nanocrystal solution diluted with deionized water (1:3 v/v) was spin‐coated at 3000 rpm subsequently annealed at 150°C for 30 min. A pre‐prepared InAs CQDs ink was spin‐casted at 3000 rpm for 1 min. Then, films were annealed at 100°C for 3 min to remove residual solvents. Next, one layer of MTA‐exchanged InAs CQDs film was further deposited by drop‐casting of one drop of InAs CQDs solution (30 mg mL^−1^ in octane) onto InAs CQDs ink film with spinning at 2000 rpm for 15 sec and treated with MTA (1 v/v% in acetonitrile) solution for 1 min, then rinsed twice with acetonitrile and once with toluene. Finally, 10 nm of MoO_3_ and 100 nm of Au were thermally deposited as the anode using a Kurt J. Lesker NANO 36. The active area of device was 3.14 mm^2^.

### 4.7 Device Characterization

The cross‐sectional SEM characterization was performed using a Zeiss GeminiSEM. All the device characterizations were performed in air under ambient conditions. Current–voltage (I–V) measurements were conducted with a Keysight Semiconductor Parameter Analyzer (B1500A) with the devices placed in a light‐shielded enclosure. The EQE characterization was conducted using a Newport Cornerstone 260 monochromator, a Thorlabs MC2000 chopper, a Stanford Research SR570 trans‐impedance amplifier, and a Stanford Research SR830 lock‐in amplifier. Calibrated Newport 818‐UV and 818‐IR photodetectors were used as references. For the noise measurements, the frequency‐dependent current noise spectrum was measured with a Variable Gain Low Noise Transimpedance Amplifier (DLPCA‐200) and a Zurich Instruments MFLI Lock‐in Amplifier. The measured room‐temperature specific detectivity (D*) was calculated according to:

$$D^{*}=\frac{R\sqrt{Af}}{i_{n}}$$

where *D* ^*^ is expressed in units of cm Hz^1/2^ W^−1^ or Jones, *A* is the active area of the photodetector, *i*_n_ is the noise current spectral density, and *f* is the noise bandwidth, here being 1 Hz. For the calculation of Figure 4e, where *i*_n_ is 1.99 × 10^−13^ A Hz^−0.5^ at 20 Hz, *A* is 0.0314 cm^2^, and *R* is 0.0377 A W^−1^ at 1300 nm; therefore the *D* ^*^ is 3.37 × 10^10^ Jones at 1300 nm at 20 Hz. For LDR measurements, a four‐channel laser (Thorlabs) at 1310 nm was used as a light source at a frequency of 7 Hz modulated by an Agilent waveform generator. The light was directed by a beam‐splitter to a Newport 818‐IG detector and the device. The current was then measured by a semiconductor parameter analyzer. To measure the 3 dB bandwidth, a nanosecond laser (520 nm) was used as the incident light, which was modulated by a waveform generator (Agilent 33220A) at various frequencies from 10 Hz to 5 MHz at 50% duty cycle. The output current was recorded with an oscilloscope.

## Author Contributions

G.K. supervised and directed the study. T.K., J.T.O., and G.K. conceptualized the study, designed the experiments, and established the narrative of the manuscript. T.K. led the synthesis, modeling, and characterization of InAs quantum dots. J.T.O. led the inkification and characterization. J.T.O. led the fabrication and characterization of the photodetectors. H.W. contributed to the device characterization. M.M. assisted with the SEM measurements. D.M. contributed to the noise measurements.

## Funding

European Research Council (ERC) under the European Union's Horizon 2020 research and innovation programme (grant agreement no. 101002306), the European Union under grant agreement No 101119489 (2DNeuralvision) and Project PID2024‐161119OB‐I00 funded by MICIU/AEI/ 10.13039/501100011033/FEDER, UE, the Fundació Privada Cellex, the program CERCA and ‘Severo Ochoa’ Centre of Excellence CEX2024‐001490‐S [MICIU/AEI/10.13039/501100011033].

## Conflicts of Interest

The authors declare no conflicts of interest.

## Supporting information




**Supporting File**: adma74004‐sup‐0001‐SuppMat.docx.

## Data Availability

The data that support the findings of this study are available from the corresponding author upon reasonable request.
