# Supplementary material for: Ligand‐Mediated Shape Engineering of SWIR InAs Colloidal Quantum Dots for Photodetector Applications
Source: Adv Mater. 2026 Jul 11;38(46):e74004. doi: 10.1002/adma.74004 (PMC13486143; doi:10.1002/adma.74004)
Supplement: Supplementary file 1 [file ADMA-38-e74004-s001.docx]

Supporting Information

Ligand-Mediated Shape Engineering of SWIR InAs Colloidal Quantum Dots for Photodetector Applications

Taewan Kim, Jae Taek Oh, Hao Wu, Marta Martos Valverde, Debranjan Mandal & Gerasimos Konstantatos*

**Figure S1.** X-ray diffraction (XRD) patterns of InAs CQDs with first excitonic absorption peaks at 920, 1200, and 1730 nm. The diffraction peaks align well with the standard zinc blende (ZB) InAs crystal structure, confirming the preservation of the zinc blende phase throughout the growth process.

**Figure S2. Particle size distribution histograms and statistical analysis of InAs CQDs**. Histograms showing the size distribution of InAs CQDs corresponding to the TEM images in Figure 1b, with first excitonic absorption peaks at 1200, 1310, 1410, 1530, and 1730 nm. The statistical summary (bottom right box plot) indicates a gradual increase in average particle size while maintaining a narrow size distribution.

**Figure S3. Absorption spectra (a), TEM images (b-e) of aliquots extracted during the InAs CQD growth reaction, and TEM image (f) of 1730 nm InAs CQD.** The TEM images b), c), d), and e) correspond to nanocrystals with first excitonic absorption peaks at 1000 nm, 1110 nm, 1340 nm, and 1550 nm, respectively. For the particles exhibiting peaks at 1000 nm and 1110 nm, the images reveal spherical or multi-faceted morphologies; consequently, their volumes are approximated using a spherical model. In contrast, the particles at 1340 nm and 1550 nm display truncated tetrahedral structures. The TEM image f) shows a less truncated structure in the largest particle. The exposed facets (yellow-colored) were identified using Fast Fourier Transform analysis. All extracted interplanar distance is ~0.36 nm, which matches the lattice spacing of the (111) plane in zinc blende InAs.

To calculate their volumes, the following equation was employed: $\frac{d^{3}-4x^{3}}{6\sqrt{2}}$ , where *d* represents the side length of the ideal tetrahedron and *x* represents the side length of the truncated portion. Based on the high-resolution TEM measurements, the 1340 nm CQDs have dimensions of d = 9.1 nm and x = 1.7 nm (four-unit), while the 1550 nm CQDs have dimensions of d = 9.3 nm and x = 2.1 nm (five-unit).

**Figure S4.** **Absorption spectra and TEM image (inset) synthesized using HMTS with (left, red line) and without (right, blue lines) chloride.**

**Figure S5. XPS spectra of as-prepared InAs CQDs films (black) and inkified InAs CQDs films processed using NOBF₄ (red) and SnCl₂ + TMAX (olive).** a) In 3d spectra. b) O 1s spectra. c) Cl 2p spectra. d) Br 3d and Sn 3d spectra of InAs CQD ink films treated with SnCl₂ + TMAX.

**Figure S6. Ultra-violet photoelectron spectroscopy (UPS) spectra of a) InAs CQDs ink films and b) MTA exchanged small-sized InAs CQDs film.**

**Figure S7.** *J−V* curves of InAs CQDs photodetector without the MTA-InAs CQDs electron-blocking layer, showing degraded rectification and high leakage current under reverse bias.

**Figure S8. Optical properties of InAs CQDs inks prepared using different ammonium halides and the corresponding performance of InAs CQDs ink photodetectors.** (a) UV–vis absorption spectra of InAs CQDs exchanged using different ammonium halides. (b) *J−V* curves and (c) EQE spectra of InAs CQDs photodetectors fabricated from InAs CQDs inks obtained using different ammonium halides.

**Figure S9.** *J−V* curves of InAs CQDs photodetector as a function of halide composition.

**Figure S10.** (a) Dark *J−V* curve of an InAs CQDs ink photodetector fabricated using InBr_3_ + NH_4_Br-passivated InAs CQDs ink. (b) UPS spectra of InBr_3_ + NH_4_Br–passivated InAs CQDs ink films. (c) Energy-level diagram of the InBr_3_ + NH_4_Br-passivated InAs CQDs ink photodetector.

**Figure S11.** Comparison of maximum EQE values as a function of wavelength for the InAs CQDs photodetectors in this work and previously reported devices synthesized using non-pyrophoric arsenic precursors. The comparison covers the SWIR spectrum ranging from 1100 nm to 1500 nm.

**Figure S12.** Frequency dependent noise power density of InAs CQDs photodetector measured with current transient fast Fourier transform and lock-in amplifier. The shot noise limit (*i*_shot_) was calculated to be 1.56 × 10^−14^ A/Hz^0.5^ using *i*_shot_ = $\sqrt{2qI_{d}}$ where q is elementary charge and *I*_d_ is the dark current at zero-bias condition and the thermal noise limit (*i*_thermal_) was determined to be 9.36 × 10^−14^ A/Hz^0.5^ using *i*_thermal_ = $\sqrt{4k_{B}T/R_{sh}}$ where *K*_B_ is the Boltzmann constant, *T* is the temperature, and *R*_sh_ is the shunt resistance. The *R_sh_* was extracted from the slope of the tangent in the low-bias region and determined to be 59.1 kΩ·cm^2^. Accordingly, the total white noise spectral density (*i*_n_) was defined as 9.49 x 10^−14^ A/Hz^0.5^($\sqrt{{i_{shot}}^{2}+ {i_{thermal}}^{2}}$). Therefore, the 1/f noise corner frequency can be estimated to be approximately 100 Hz.

**Figure S13.** Frequency dependent specific detectivity at 1300 nm of InAs CQDs photodetector.

**Figure S14.** a) UV–vis absorption spectrum of the 1360 nm InAs CQDs ink solution. b) Dark *J−V* characteristics and c) corresponding EQE spectra of the InAs CQDs photodetector fabricated using 1360 nm InAs CQDs ink.

**Figure S15.** a) UV–vis absorption spectrum of the 1415 nm InAs CQDs ink solution. b) Dark *J−V* characteristics and c) corresponding EQE spectra of the InAs CQDs photodetector fabricated using 1415 nm InAs CQDs ink.

**Figure S16.** Response time of InAs CQDs photodetector with a device area of 3.14 mm^2^ at 0 V.

**Figure S17.** The operational stability of InAs CQDs SWIR photodetector, showing measured dark current and photocurrent under 1300 nm illumination.
